# A Comprehensive Strategy to Mitigate Institutional Maternal Mortality: Lessons From a Quality Improvement Initiative in Brazilian Maternity Hospitals

**DOI:** 10.9745/GHSP-D-24-00130

**Published:** 2025-12-31

**Authors:** Paulo Borem, Andrea Keiko Fujinami Gushken, Ana Paula Gushken, Rodolfo de Carvalho Pacagnella, Ademir Jose Petenate, Paula Tuma, Livia Sanches Pedrilio, Santiago Nariño, Pierre Barker, Claudia Garcia de Barros, Sebastian Vernal

**Affiliations:** a Institute for Healthcare Improvement, Cambridge, MA, USA.; b Departamento de Ginecologia e Obstetrícia, Universidade de Campinas, Campinas, SP, Brazil.; c Escritório de Excelência, Hospital Israelita Albert Einstein, São Paulo, SP, Brazil.

## Abstract

**Introduction:** Many pregnancy-related deaths can be avoided if health care workers reliably provide prompt, evidence-based care during the final stages of pregnancy and delivery. We report the impact on the institutional maternal mortality ratio (iMMR) of a quality improvement (QI) initiative that focused on establishing timely and reliable obstetric care in public Brazilian hospitals for the primary causes of maternal deaths.

**Methods:** The QI initiative used a quasi-experimental time-series design implemented in 19 Brazilian maternity hospitals comparing 3 periods: baseline (January 2018 to November 2019), implementation (December 2019 to March 2021), and post-implementation (April 2021 to September 2021). We used a sequential approach, referred to as the “4Rs” (Recognize, Rescue, Reassess, and Refer), with the Modified Early Obstetric Warning Score (MEOWS) as a key tool, to identify clinical deterioration and implement care bundles directed at the management of 3 principal life-threatening conditions around the time of birth: postpartum hemorrhage, sepsis, and hypertensive disorders of pregnancy (HDPs). Clinical staff received tailored training and support within a structured learning system that brought multiple teams together to rapidly test and implement evidence-based changes.

**Results:** Comparing the baseline with the implementation period, run charts detected an overall reduction of 34.2% in all causes of maternal deaths (from 83.7 to 55 deaths per 100,000 live births). Additionally, the iMMR due to the 3 analyzed life-threatening conditions decreased by 60.9% between baseline and the combined implementation and post-implementation periods (from 44.1 to 17.2 deaths per 100,000 live births); postpartum hemorrhage-related deaths were reduced by 72.9% (from 11.7 to 3.17 deaths per 100,000 live births) and sepsis-related deaths were reduced by 100% (from 20.4 to 0 deaths per 100,000 liv births). No changes by HDP-related deaths were observed. There was a 178% increase in iMMR by all causes (mainly attributed to COVID-19) during the post-implementation period, but deaths related to life-threatening conditions either remained stable or decreased even further.

**Conclusion:** Optimization of the steps in a care sequence and reliable implementation of care bundles directed at the principal life-threatening conditions around the time of birth, using QI, tailored training, and the MEOWS tool, seem to be feasible and promising approaches for reducing iMMR.

## Abstract (precis)

Maternal deaths can be prevented through quality improvement and job instruction methods that enhance the early detection and management of the primary life-threatening conditions contributing to institutional maternal mortality.

## Teaser

**Key Findings:**

- The “4Rs” clinical sequence—Recognize, Rescue, Reassess, and Refer—implemented with a quality improvement framework and tailored Job Instruction training is an effective approach to managing the principal life-threatening conditions responsible for institutional maternal mortality.
- The intervention was associated with a 34.2% decrease in the institutional maternal mortality ratio (iMMR) when comparing the baseline to the implementation period in 19 public Brazilian maternity hospitals.
- While maternal deaths increased after the quality improvement implementation period due to a staggering number of COVID-19 deaths during the pandemic, deaths from the 3 targeted life-threatening conditions either remained stable or decreased even further, suggesting the sustainability of the established processes during the initiative.

**Key Implication:**

- Structured, systematic, and auditable methodologies to redesign the care process can help improve maternal care outcomes. This approach can inform strategies to reduce iMMR worldwide if replicated and scaled in different settings.

## BACKGROUND

Circumstances leading to maternal death are complex and multifactorial, but up to 80% of peripartum deaths are thought to be preventable.^1^ Across the globe, the leading causes of mothers’ death are postpartum hemorrhage (27%), hypertensive disorders of pregnancy (HDPs) (14%), sepsis (11%), complications of abortion (8%), embolism (3%), and other direct causes such as obstructed labor and complications of delivery (10%).^2^ These are the same leading causes observed in Brazil and other low- and middle-income countries (LMICs).^3^^,^^4^ HDPs, including eclampsia and preeclampsia, are a significant contributor to mortality in Latin America and the Caribbean, contributing to 22% of all maternal deaths in the region.^4^

Quality improvement (QI) is a systematic, data-driven approach to enhance health care processes and outcomes through iterative testing and refinement. QI methods can help to strengthen the delivery of reliable interventions that are effective, sustainable, and cost-effective.^5^^,^^6^ When used to support clinical interventions, QI methods have successfully improved maternal health outcomes in both high- and low-income nations.^7^^–^^9^

A care bundle is a set of evidence-based practices that, when implemented together, improve patient outcomes more effectively than when applied individually. Care bundles used to manage an emergency are well-recognized strategies to improve adherence to clinical standards to manage life-threatening conditions, reducing the time between recognition and treatment.^10^^,^^11^

Despite numerous advances in reducing maternal death, LMICs are far from reaching the United Nations Sustainable Development Goals related to maternal and newborn outcomes. To address this challenge in Brazil, the Institute for Healthcare Improvement, in collaboration with the Albert Einstein Hospital and MSD Brazil, carried out a QI initiative to reduce the institutional maternal mortality ratio (iMMR) using a framework developed by the Institute called the Breakthrough Series Collaborative (BTS). BTS is a structured, short-term learning system that brings together multiple teams to rapidly test and implement evidence-based changes, closing the gap between best practices and daily operations to achieve breakthrough improvements in quality and outcomes. This article presents the learnings and results of this QI initiative.

## METHODS

### Context

Between 1990 and 2013, the national maternal mortality ratio (MMR) in Brazil dropped by 55%. Since then, however, MMR in Brazil has been unchanged, ranging generally between 55 and 60 maternal deaths per 100,000 live births,^3^^,^^12^ with the exception of during the height of the COVID-19 pandemic (March 2020 to May 2021), when maternal mortality increased by more than 70%.^12^^,^^13^

In Brazil, maternal death is primarily associated with the delay in recognizing clinical deterioration at the health facility level and the reliability of providing effective and standardized care.^14^^,^^15^ Well-designed processes to recognize clinical deterioration and perform immediate clinical actions are crucial to prevent pregnancy-related mortality.^14^^,^^15^

The circumstances that lead to maternal death are complex and multifactorial, including factors related to inequity and racism. For example, by 2018, more than a half of all maternal deaths occurred among Black women, who are twice as likely to die during pregnancy and childbirth than White women.^16^^,^^17^ Regrettably, during the pandemic, these inequities were even more evident. Black women accounted for 1,095 of the pregnant and postpartum women who died from COVID-19, representing 54% of this group by March 2022.^12^

### Implementation Approach

Our QI initiative utilized the Model for Improvement,^5^ a widely recognized framework developed by the Institute for Healthcare Improvement, that guides teams to set specific improvement aims, identifies processes and outcomes to measure, and develops change ideas to test, using rapid Plan-Do-Study-Act (PDSA) cycles. Teams were supported to identify system inefficiencies, prioritize interventions, and foster a culture of continuous improvement through collaboration and shared learning. The approach is grounded in evidence-based practice and integrates both quantitative and qualitative data to ensure a comprehensive understanding of system performance and outcomes. The teams were connected through a series of virtual and face-to-face meetings using the BTS methodology, which provided a platform for the teams to learn, collaborate, and rapidly disseminate best practices.^18^

Before launching, the BTS governance team convened a meeting of implementers, clinical experts, and hospital leaders to create consensus about the initiative’s aim, clinical intervention, and implementation strategy. The outputs of the meeting included (i) an aim to reduce the iMMR by 36%, with focus on the 3 most prevalent life-threatening conditions, (ii) definition of the clinical intervention as the “4Rs” sequence, described below, and care bundles for the life-threatening conditions, and (iii) agreement on the implementation approach that addressed a set of system enablers, using QI methods and tailored training of the clinical staff.^19^

At the first learning session, we engaged hospital teams to identify the local environmental conditions and capabilities that support the teams to implement the clinical changes. These primary drivers of improved survival and associated ideas for improvement are summarized in Table 1.

**TABLE 1.** Primary Drivers of Improved Women’s Survival Around Birth and Associated Changes the Participating Hospitals Tested

**Primary Driver (what to do)**	**Changes (how to do)**
Create a reliable process to identify patients with suspected LTCs.	Use MEOWS in addition to the standard screening tool for all patients (MEOWS + screening tool).
	Add 2 questions for women with MEOWS (MEOWS ≥ 4 or a single parameter score of 3): Can the patient have an infection (suspicion of sepsis)? Can the patient have HDP?
	Create alert systems for the entire team when suspected LTC is identified through MEOWS ≥ 4 or a score of 3 on any parameter + yes in 1 of the 3 questions or blood loss ≥500 mL vaginal birth or ≥1,000 mL for cesarean delivery.
Provide safe, reliable, and equitable care for all patients at admission, antepartum, postpartum, and discharge.	Ensure the timely rescue of all patients with suspected LTC by competent and trained teams to apply the care bundles.
	Reassess all patients according to bundle recommendations using MEOWS.
	Ensure to escalate the care of all patients with any LTC.
	Train teams to adapt the care considering social and racial inequalities.
Develop engaged care teams with knowledge of how to deal with LTCs, considering social and racial inequalities.	Train teams from all departments to provide clinical assistance to patients with LTCs.
	Promote training in the Model for Improvement.
	Build capacity (process/people) in the organization to collect and publish measures disaggregated by race reliably.
Develop a culture of safety and continuous improvement.	Create highly effective interdisciplinary teams (huddles, SBAR for communication, adverse events analysis).
	Engage leaders to promote transparency, fair culture, and quality of care as a high organizational priority.
Ensure that service is co-designed with patients, families, and community.	Engage patients/family/community to co-design care processes.
	Improve transparency in communication with patients/family/community.

Abbreviations: LTCs, life-threatening conditions; MEOWS, Modified Early Obstetric Warning Score; HDP, hypertensive disorders of pregnancy; SBAR: Situation, Background, Assessment, Recommendation.

The implementation intervention started with a 2-day face-to-face learning session in November 2019. Due to the COVID-19 pandemic, all subsequent quarterly face-to-face 2-day sessions between December 2019 and March 2021 were replaced with 1-hour monthly virtual sessions. During the in-person and virtual sessions, QI teams received training on QI methods; learned how to design initial tests of change using PDSA cycles to increase the percentage of patients with a calculated Modified Early Obstetric Warning Score (MEOWS) and the use of the care bundles; and shared experiences of successes and challenges during implementation. QI teams comprised an executive leader, obstetrician, and obstetric nurse.

Between the 1-hour monthly virtual learning sessions, we offered one-to-one support to the hospitals upon request. We asked each hospital to send a monthly report including a description of the tests of changes, changes implemented, and updates of the process and outcomes measures. Improvement coaches provided QI teams with feedback mentoring based on the data and information included in these reports. To coach leaders to support and enable change in their systems, the leadership of the hospitals participated in 2 separate virtual learning sessions explicitly designed to convey the crucial role of leadership (e.g., support for the initiative, understanding of the methods, removal of barriers, and presence at the front line, among other topics),^20^ and to advance equitable care.

### Job Instruction

Training Within Industry,^21^ a proven methodology for standardizing and improving work processes, has gained traction in health care settings after demonstrating its effectiveness across various industries. Training Within Industry provides a structured framework for training individuals to perform tasks correctly, safely, and efficiently.

In this initiative, we utilized Job Instruction,^22^ one of the core components of Training Within Industry, to enhance the implementation of care bundles for maternal health. Job Instruction involves breaking down complex tasks into key elements, including important steps, key points, and their underlying rationale. By providing this structured approach, we supported health care providers to practice these elements in a standardized way, fostering consistency and quality in performance across all maternal care processes.

Job Instruction requires the teams to create Job Breakdown Sheets of the most important processes containing the important steps, key points (related to safety and best performance), and the reasons of each key point.^23^ With the Job Breakdown Sheets ready, the instructor will train the front line, one-by-one, using the 4 steps of training:

1. Prepare the worker
2. Present the process
3. Try out performance
4. Follow up

Clinical skills require a competency-based approach with a strong emphasis on skill acquisition. While Job Instruction is valuable for hands-on tasks, its effectiveness is maximized through individualized instruction and practical application. Ideally, this involves one-on-one training, allowing for personalized feedback and repetition. Furthermore, conducting the training in a simulated environment or the actual clinical setting where the procedure takes place enhances skill transfer and confidence.

In our case, the BTS team trained virtually 1–2 health care workers per hospital, providing a foundation in the Job Instruction methodology. The subsequent face-to-face training at each hospital offered by the health care professionals was crucial for ensuring competency in hands-on clinical skills. This approach allowed for direct observation, individualized feedback, and practice in the actual clinical environment, ultimately leading to improved skills to care for patients.

### Study Design

We undertook a quasi-experimental time-series study to determine the effectiveness of the QI initiative to improve standard clinical surveillance and care and the impact of this on iMMR in public maternity hospitals across Brazil. To evaluate changes in the iMMR, we analyzed data collected from the 19 participating organizations during 3 periods:

- Baseline (January 2018 to November 2019)
- Implementation (December 2019 to March 2021)
- Post-implementation (April 2021 to September 2021)

This article follows the structure of Standards for Quality Improvement Reporting Excellence (SQUIRE 2.0).^24^

### Settings and Participants

All 28 public hospitals that had participated in another Brazilian QI Collaborative to reduce high cesarean delivery rates were invited to join the BTS initiative.^25^ Of these, 20 institutions volunteered to join the new initiative. In addition, 1 hospital with a high iMMR in the north of Brazil that had not participated in the other QI Collaborative asked to join and was also included in the BTS.

### Clinical Intervention

This initiative focused on the 3 most common causes of maternal death: postpartum hemorrhage, sepsis, and HDPs. “Failure to rescue” is the failure or delay in recognizing and responding to a hospitalized patient experiencing complications from a disease process or medical intervention,^26^ and the concept has been broadly applied to obstetric settings where an estimated 40% of obstetric deaths have been attributed to this breakdown in clinical care.^27^

#### The 4Rs

We structured the clinical activities according to a sequence of 4 steps that recognizes, rescues, reassesses, and refers (“4Rs”) patients to the most appropriate level of care after clinical deterioration, applied to every woman admitted to each of the hospitals for obstetric care:

- **Recognize:** For sepsis and HDPs, we used MEOWS^28^ for timely detection of clinical deterioration. This score was used at the time of first contact with the patients in each transition of care and to reassess the patient’s clinical condition after the second R (Rescue). MEOWS has been used extensively, including in Brazilian studies, to support efforts to reduce maternal mortality.^29^^–^^33^ For postpartum hemorrhage, we used blood loss ≥500 mL for a vaginal birth and ≥ 1,000 mL for cesarean delivery as the primary trigger to initiate a rescue response,^34^ as this preceded clinical deterioration.
- **Rescue:** A response to early clinical deterioration. In this initiative, we used care bundles and tailor-made clinical interventions to manage hemorrhage. Additionally, we used care bundles specifically designed to manage any suspicion of HDPs. The bundle for treating suspected sepsis adheres to the standards recommended by the 2018 Surviving Sepsis Campaign guidelines.^35^ For postpartum hemorrhage and HDPs, our team of clinical experts selected evidence-based interventions after a comprehensive literature review and the BTS team developed the bundles using the Model for Improvement.
- **Reassess:** Reassess the effects of the rescue using MEOWS.
- **Refer:** Escalate the care to the most appropriate level within or outside the hospital.

The 4Rs approach was a modification and expansion of a sequence of clinical actions originally described for the management of obstetric-associated hemorrhage.^36^ Before this initiative, none of the hospitals had a standardized way to manage the 3 life-threatening conditions with the interventions based solely on the decision of the physician during the event.

#### Care Bundles

The care bundles were based on the concept originally proposed by the Institute for Healthcare Improvement^11^ and widely adopted in obstetric practice^36^ that a small set of clinical interventions applied together, directed at a specific clinical problem, is a more effective clinical strategy than piecemeal application of those care elements. During this BTS, these evidence-based clinical bundles were introduced to hospitals that did not previously have standardized evidence-based protocols to manage the 3 life-threatening conditions. The ultimate use of the bundle elements was left to the discretion of the attending physician during the event, depending on the patient’s unique needs. The sequential approach to diagnosis and treatment was modified from a previously reported approach to management of obstetric-associated hemorrhage.^36^

The care bundles for postpartum hemorrhage, sepsis, and HDPs are shown in Table 2. Compliance with the bundle was monitored through regular audits and results were used to guide iterative refinements and support team learning throughout the improvement process.

**TABLE 2.** Care Bundles for Postpartum Hemorrhage, Sepsis, and Hypertensive Diseases of Pregnancy

**Care Bundle**	**Items in the Care Bundle**
PPH first response care bundle^a^	TROM: Tranexamic Acid, *Reposição volêmica* (IV fluids), Oxytocic drugs, and Massage (uterine)^b^ Treat each of the 4 “Ts” accordingly (tone, trauma, tissue, and thrombin)
PPH second response care bundle^c^	Calculate shock index Move patient to operation room Treat each of the 4 “Ts” accordingly Consider blood transfusion
HDPs care bundle	Collect laboratory tests for preeclampsia Magnesium sulfate loading dose Magnesium sulfate maintenance dose Control of blood pressure and respiratory rate Recalculate MEOWS Treat high blood pressure
Sepsis care bundle	Measure lactate level Obtain blood cultures prior to administration of antibiotics Administrate antibiotics Rapidly administer 500 mL IV solution Apply vasopressor after IV fluid to maintain mean arterial pressure ≥65 mmHg

^a^ For blood loss ≥500 mL (vaginal birth) or ≥1,000 mL (cesarean delivery), regardless of altered vital signs.

^b^ After this BTS, a group or researchers published an article proposing E-MOTIVE^36^ instead of TROM. The elements of the bundle are the same, but arranged in a different order, which changes the acronym.

^c^ After applying the first response bundle and blood loss persists.

Abbreviations: BTS, Breakthrough Series; HDPs, hypertensive disorders of pregnancy; MEOWS, Modified Early Obstetric Warning Score; PPH, postpartum hemorrhage.

In addition to care bundles, we also proposed 3 actions to prevent postpartum hemorrhage: assess hemorrhage risk, quantify blood loss during delivery, and adminster 10 IU oxytocin after delivery. Blood was collected using a drape during vaginal delivery or a suction canister during a cesarean delivery. Blood-soaked items like pads and sponges were weighed, and their dry weight was subtracted to calculate how much blood they had absorbed. The total blood loss was then determined by adding the collected blood to the amount calculated from the soaked items.

**FIGURE 1:**
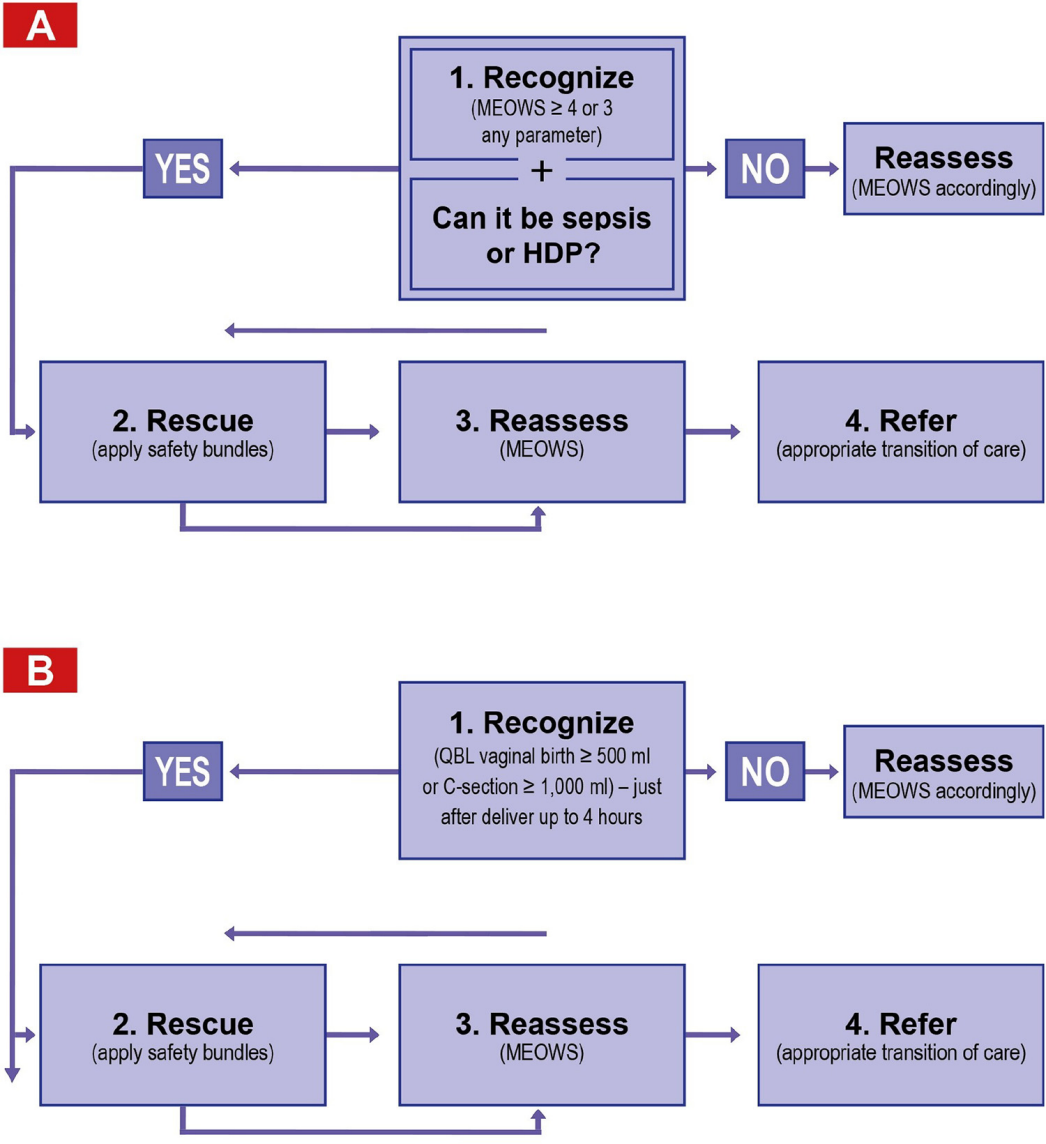
The “4 Rs” Flowchart for Sepsis and Hypertensive Disorders of Pregnancy (A) and for Hemorrhage (B) Abbreviations: C-section: cesarean delivery; MEOWS: Modified Early Obstetric Warning Score; QBL, quantity blood loss.

For blood loss >1,000 mL in a vaginal birth or >1,500 mL in a cesarean delivery, traditional care bundles are not suitable. The expert panel recommended that obstetricians and gynecologists utilize a more individualized approach to invasive procedures, tailoring them to each patient’s specific needs and clinical circumstances. These procedures may include a hysterectomy, the use of an intrauterine balloon, compressive sutures, and vascular embolization. The integration between the “4Rs” and care bundles’ triggers is summarized in Figure 1.

All participating hospitals were required to implement at least 1 care bundle for 1 of the 3 life-threatening conditions. The choice of life-threatening condition was determined by the preferences of the QI teams at each hospital; however, all hospitals had access to all care bundles. Regardless of the chosen life-threatening condition to focus on, each hospital was required to implement the proposed actions to prevent postpartum hemorrhage.

The care bundle for postpartum hemorrhage was divided into 2 bundles designed to be applied sequentially. The first response care bundle was applied when blood loss was ≥500 mL and ≤1000 mL for vaginal birth and ≥1000 mL and ≤1500 mL for cesarean delivery. The second response care bundle was applied if the patient continued to bleed after the application of the first response care bundle. If bleeding persisted despite the application of both care bundles, the treatment was determined by the clinical judgment of the care team.

#### Equity and Anti-Racism Training

Recognizing the profound impact of racial disparities on maternal health outcomes, we incorporated anti-racism training as a critical component of this QI initiative. Evidence demonstrates a disproportionately high rate of maternal mortality among Black women in Brazil, highlighting the urgent need to address systemic inequities within the health care system.^12^ To mitigate the influence of implicit bias and promote equitable care for all patients, we conducted virtual anti-racism training sessions for hospital leaders between October 2020 and February 2021. These sessions aimed to raise awareness of racial disparities in maternal health and empower leaders to implement strategies that counter race-based inequities in their respective institutions. A detailed description of this intervention and its impact will be published in a separate manuscript.

#### Enabling the Environment for Change

Meetings between experts and the hospital-based leadership team addressed the system enablers (all necessary drugs, materials, time allocated, and equipment) required for the frontline team to carry out the new standard of care (the “4Rs,” MEOWS, and care bundles). They guided leadership behaviors (e.g., walk rounds and devolved leadership approaches). Leadership buy-in was sought to allow more autonomy for the nurses to trigger and apply the care bundles previously approved by the obstetricians.

### Measurement

QI teams collected the following data from hospital records: the number of births, maternal mortality by cause, the use of MEOWS, the number of women diagnosed with potential life-threatening conditions, and the number of live births.

QI teams provided baseline data on the number of live births and deaths 12 months prior to the intervention. For all other measures, baseline data was established by analyzing hospital data for common and special cause variation during the intervention according to Shewhart.^37^ Except for maternal mortality, all other measures were newly implemented by the hospitals as part of this QI initiative.

To be compliant with the care bundles, the clinical teams had to have applied all elements of the bundle (“all or nothing” concept),^11^ but we also measured the percentage of patients receiving each element of the bundle.

For process measures, we created a spreadsheet for each hospital with instructions on collecting and reporting data. We recommended a random sample of at least 20 patients monthly for each process measure. The way the QI teams collected process measures varied. Some collected the process measures by reviewing patients’ charts. Other hospitals collected process measures while the care was happening. In both cases, the QI teams used the tool created by our team to collect data. The hospital QI team assigned 1 person to collect data and enter it into the online platform. During the implementation period, the QI team at each site shared their de-identified aggregate monthly measures using online data entry (Simple QI^®^). All measures’ formulas are described in the Supplement Table.

For this initiative, we used aggregated data from the units to track changes in iMMR over time. This approach was adopted because every hospital (despite focusing on a specific bundle) had access to the complete set of bundles. Subgroup analysis of hospitals applying improvement to individual bundles was not done because of sample size limitations.

### Analysis

We used monthly run charts analysis^38^ to detect changes from the baseline for all causes of death and the life-threatening condition. To detect significant changes in performance from the baseline, we extended the median baseline values into the implementation and post-implementation period and applied statistical process control rules to detect special cause variation. The causes of maternal deaths, including COVID-19 deaths, are presented in a table, and we used a scatter plot chart to correlate the percentage of patients with MEOWS score calculated and the iMMR. Minitab^®^ v18 (USA) was used for all statistical analyses.

### Ethical Considerations

For this article, access to the BTS database was approved by the Research Ethical Board of Albert Einstein Hospital. The database has QI process measures, aggregated and de-identified, and does not exhibit any data referring to or mentioning the patients involved, compliant with local data protection laws. The present article does not present any data that could identify the facilities.

## RESULTS

Of the 21 hospitals that joined the BTS at the start of this initiative, 1 hospital never provided data and was excluded from the study, and another hospital dropped out.

We included the remaining 19 maternity hospitals participating in the initiative and contributing data for the analysis. All 19 maternity hospitals provided data on iMMR consistently for the entire baseline, implementation, and post-implementation period. Most institutions were in the southeastern region of Brazil (63%), and 7 belonged to Brazil’s north/northeastern region (37%). Table 3 describes the characteristics of the participating institutions.

**TABLE 3.** Characteristics of the Participating Institutions, by the Life-Threatening Condition Chosen for Improvement

**Life-Threatening Condition to Improve**	**No. of Institutions**	**Type of Administration** ^a^	**Median Live Births Between April 2018 and March 2021 (Range)**
Postpartum Hemorrhage	7	3 public (1 teaching) 3 philanthropic 1 social organization	11,813 (3,814–19,377)
Hypertensive Diseases of Pregnancy	8	4 public (1 teaching) 2 philanthropic 2 social organization	7,055.5 (3,903–12,528)
Sepsis	4	4 public (1 teaching)	14,269 (5,828–27,597)

^a^ Public hospital: Health care facility that is owned, funded, and operated by the government or a government agency at the local, regional, or national level; Teaching hospital: a medical facility that is affiliated with a medical school or university and is actively involved in the training and education of medical students, interns, residents, and other health care professionals; Philanthropic hospital: health care institution that operates with a primary focus on providing medical services and care as part of its charitable mission; Social organization: health care institution managed by an organized group of individuals who come together to pursue common goals, share values, and establish structured relationships to achieve specific purposes.

During the implementation period of the intervention, efforts were made to enhance clinical skills and redesign the care model in the participating maternity hospitals. As demonstrated in Table 4, there was a notable change in care practices and the obstetric care environment favorable for iMMR reduction.

**TABLE 4.** Changes Documented in the Hospitals Before and After the BTS, by the “4Rs” Clinical Steps

**Clinical Step (The 4Rs)**	**Before the BTS**	**After 17 months of the BTS**
**Recognize**	MEOWS not included in existing tools and protocols for triage and vital signs monitoring in most participating hospitals	MEOWS was adopted across the hospitals.
	Inconsistency among HCWs in interpreting the urgency to act when faced with abnormal vital signs and managing clinical situations	The HCW team is well-trained on tools and protocols using Job Instruction/TWI methods.
	Delayed critical interventions and compromised patient safety due to absence of a clear threshold for action	A clear threshold to act: if MEOWS score of 4 or greater, or a score of 3 in any single parameter, the HCW needs to ask if there is suspicion of sepsis or preeclampsia. With hemorrhage, the threshold is blood loss.
**Rescue**	Predominant obstetrician-based decision-making	Team-based care and standardized.
	Decision-making variable depending on HCW background experience and preferences	Leadership and frontline team trained in using data for decision-making and rapid cycle improvement.
	No QI capability to reflect on performance and improve systems of care	HCWs use improvement sciences to systematically improve the quality of care.
	Significant variations in clinical care due to unclear protocols and clinical decision-making	Collaborative agreement on best practices and evidence-based interventions reduces care variation.
**Reassess**	No standard tools or process to reassess the patients after interventions	MEOWS is the gold standard for reassessing patients after implementing care bundles or clinical interventions. There is a clear process for communicating patient status to the care team.
**Refer**	No explicit standard tool during the transition of care	MEOWS is undertaken for all patients requiring care transitions with a clear recommendation to act.
	No standard referral pathways	Streamlined referral pathways to ensure optimal patient care.

Abbreviations: BTS, Breakthrough Series; HCW, health care worker; MEOWS, Modified Early Obstetric Warning Score; QI, quality improvement; TWI, Training Within Industry.

Across the 19 hospitals, the implementation of quantifying blood loss rose from 0% to 95%, and the administration of 10 IU of oxytocin after delivery rose from 78% to 98% (data not displayed). In the case of sepsis, the percentage of patients for whom health care providers reliably used the care bundle increased significantly from 42% to 82%.

**FIGURE 2:**
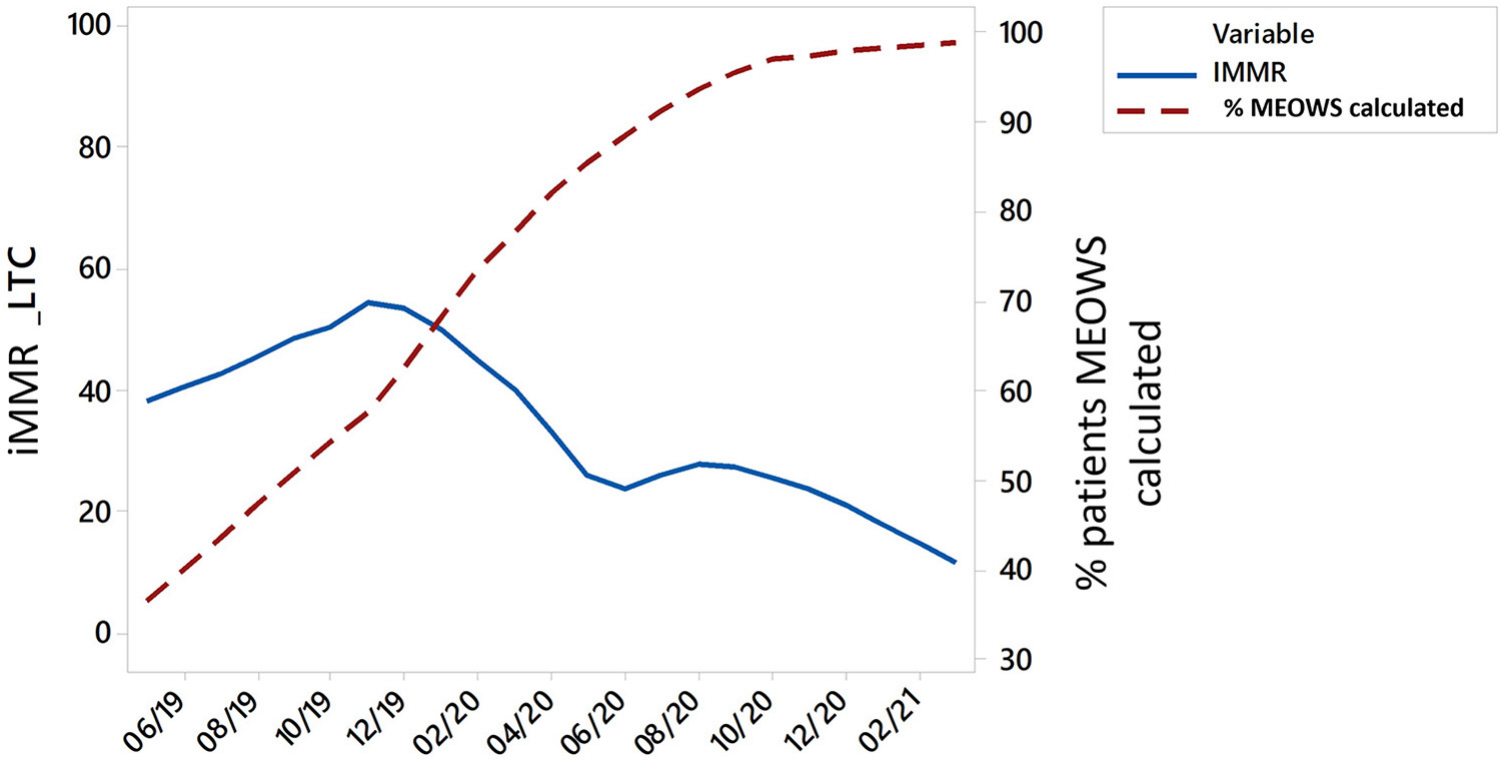
Correlation Between Implementation of MEOWS and the iMMR From Life-Threatening Conditions, June 2019 to March 2021 Abbreviations: iMMR, institutional maternal mortality ratio; LTC, life-threatening condition; MEOWS, Modified Early Obstetric Warning Score.

After the intervention began, hospitals were instructed to track and report the percentage of patients with MEOWS scores recorded. Just after the hospitals started testing and implementing MEOWS, 61% of patients had scores documented. This percentage steadily increased throughout the implementation period, ultimately stabilizing at 98%. We saw an inverse relationship over time between the reduction of iMMR and the increased adoption of MEOWS (Figure 2).

We estimate that between December 2019 and September 2021, the QI interventions prevented approximately 33 maternal deaths due to life-threatening conditions and other causes, except COVID-19 (Table 5). However, the number of live births was higher during the baseline period than the implementation and post-implementation periods. This could lead to an argument that the exposure to risk was lower during and after implementation, which would naturally result in fewer deaths. A closer examination of the data, however, reveals a different story. When we calculate the iMMR by dividing the number of deaths during the baseline period by the number of live births, we arrive at an iMMR of 76.4 (102/133,471 X 100,000 live births). In contrast, when we perform the same calculation for the implementation and post-implementation periods, the iMMR is significantly lower at 27.3 (33/120,916 X 100,000 live births). Therefore, the observed reduction in the number of deaths cannot be attributed to the difference in live births during the baseline period compared to the implementation and post-implementation periods.

**TABLE 5.** Number of Deaths per Period and the Estimated Number of Deaths Prevented, by Cause of Death

	**Number of Deaths per Period**			**Estimated Number of Deaths Prevented** ^a^
**Cause of Death**	**Baseline:** **January 2018 to November 2019**	**Implementation:** **December 2019 to March 2021**	**Post-Implementation:** **April 2021 to September 2021**	**During and After Implementation: December 2019 to September 2021**
Hypertensive Disorders of Pregnancy	15	11	5	0
Postpartum hemorrhage	15	3	1	11
Sepsis	26	8	0	18
Others (all causes except the 3 analyzed life-threating conditions)	46	31	10	5
**Total (3 life-threating conditions and other, except COVID-19)**	**102**	**53**	**16**	**4**
**Live Births**	**133,471**	**120,916**		

^a^ To estimate the number of deaths averted, we employed the following method: initially, we established a baseline by gathering data on maternal mortality within a defined timeframe preceding the intervention. Subsequently, we calculated the baseline maternal mortality rate by dividing the number of maternal deaths by the number of live births during that period. Using this rate, we projected the anticipated number of maternal deaths, multiplying the rate by the number of live births each month, commencing with the first month after the baseline period. This projection illustrated the expected number of maternal deaths had the intervention not been implemented. Throughout the project, we meticulously collected the actual number of maternal deaths each month. By subtracting the actual number of deaths from the projected number, we determined the difference, representing the number of deaths averted.

**FIGURE 3:**
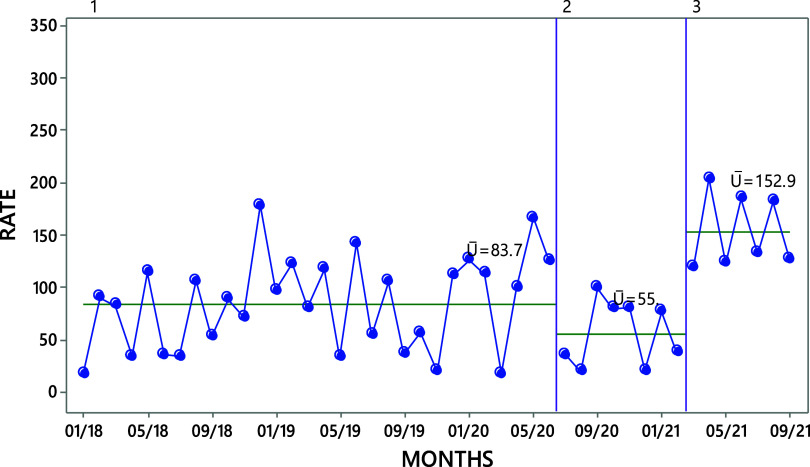
Run Chart of the Aggregated iMMR From All Causes of Death in the 19 Participating Hospitals, January 2018 to September 2021^a^ ^a^ December 2019 to March 2021 represents the implementation period of the quality improvement initiative. Abbreviations: iMMR, institutional maternal mortality ratio.

Comparing the baseline to the implementation period, analysis of run charts detected a 34.2% reduction in the all-cause iMMR (from 83.7 to 55 deaths per 100,000 live births). However, there was a sharp increase of 178% between the implementation and post-implementation period, from 55 to 152.9 deaths per 100,000 live births, with 70% of the deaths attributed to COVID-19 (Figure 3).

**FIGURE 4:**
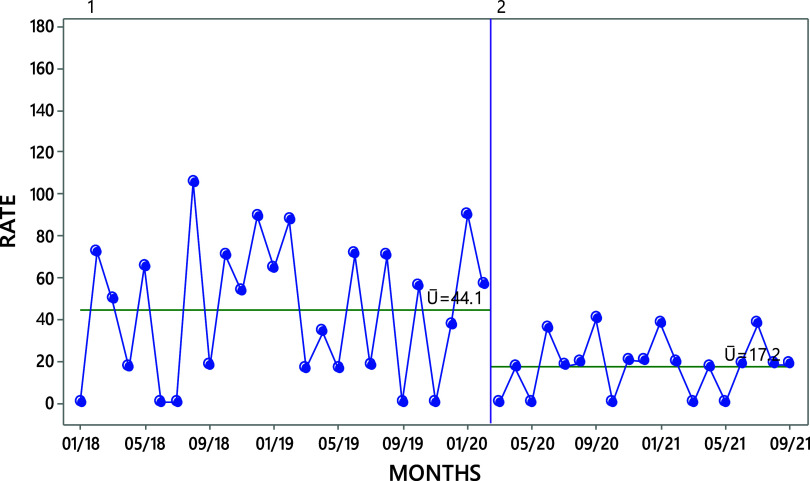
Run Chart of the Aggregated iMMR Due to Life-Threatening Conditions^a^ in the 19 Participating Hospitals, January 2018 to September 2021^b^ ^a^ Life-threatening conditions comprised postpartum hemorrhage, sepsis, and hypertensive disorders of pregnancy. ^b^ December 2019 to March 2021 represents the implementation period of the quality improvement initiative while April to September 2021included the post-implementation period. Abbreviations: iMMR, institutional maternal mortality ratio.

Comparing the baseline to the combined implementation and post-implementation period, the iMMR due to life-threatening conditions decreased by 60.9%, from 44.1 to 17.2 deaths per 100,000 live births (Figure 4). The iMMR due to postpartum hemorrhage decreased by 72.9%, from 11.7 to 3.17 per 100,000 live births, while the iMMR from sepsis decreased by 100%, from 20.4 to 0 deaths per 100,000 live births (data not displayed). The number of deaths did not decrease for HDPs.

Table 6 shows the variation in percentage points in the care bundle practices suggested by the QI initiative. Data for the postpartum hemorrhage second response care bundle are not included because none of the hospitals collected the related data.

**TABLE 6.** Variations in Care Bundle Practices Among Participating Hospitals, May 2019 to October 2021

**Care Bundle**	**Items in the Care Bundle**	**Reporting Hospitals (N)**	**Initial Control-Line (%)**	**Final Control-Line (%)**	**Variation (percentage points)**
**MEOWS**	N/A	19	78	98	**+20**
**PPH prevention actions**	Assess hemorrhage risk	16 of 19^b^	92	92	0
	Quantify blood loss		57	90	**+33**
	10 IU oxytocin after delivery		80	99	**+19**
**PPH first response care bundle**	Tranexamic acid	6 of 7	63	90	**+27**
	IV fluids		76	84	**+8**
	Oxytocic drugs (higher dose)		76	87	**+11**
	Uterine massage		74	89	**+15**
**HDP care bundle**	Magnesium sulfate (loading dose)	4 of 8	86	86	0
	Magnesium sulfate (maintenance dose)		54	75	**+21**
	Treat high blood pressure^a^		87	87	0
**Sepsis care bundle**	Measure lactate level	3 of 4	42	88	**+46**
	Obtain blood cultures prior to administration of antibiotics		84	84	0
	Administer antibiotic		92	92	0
	Rapidly administer 500 ml IV solution		53	93	**+40**
	Apply vasopressor after IV fluid to maintain mean arterial pressure ≥ 65 mmHg		91	91	0

Abbreviations: HDP, hypertensive disorders of pregnancy; MEOWS, Modified Early Obstetric Warning Score; N/A, not applicable; PPH, postpartum hemorrhage.

^a^ Hydralazine was the antihypertensive/vasodilator available in the participating hospitals.

^b^ The reporting hospitals varied from 5 to 16, reaching 16 in the last 3 months of the BTS.

## DISCUSSION

This BTS initiative presents a feasible QI strategy for decreasing iMMR in public hospital settings in a developing country. This BTS addressed the 3 leading maternal causes of death found by the WHO: postpartum hemorrhage, sepsis, and HDPs. After the QI intervention, we observed a reduction in deaths related to postpartum hemorrhage and sepsis, but not HDPs, resulting in a lower aggregate iMMR. The durability of the reduction in deaths from hemorrhage and infection was proved despite the disruptions associated with the COVID-19 pandemic. These findings reinforce the impact and sustainability of the redesign of obstetric care in hospitals that were delivered using a combination of QI and targeted trainings.

Despite efforts to reduce maternal deaths in Brazil, MMR has remained high and relatively unchanged since 2013.^3^^,^^12^ Our results support the calls for expanding traditional strategies that focus on clinical training, infrastructure, medical equipment, medications, and human resources to include more engaging training methods for clinical bundles^39^ combined with QI approaches^40^ to implement evidence-based care more reliably.

The core achievement of our initiative was the successful implementation of the clinical intervention to reduce iMMR from the major life-threatening conditions using the “4Rs,” which included an adaptation of the “failure to rescue” concept^26^ applied to the resource-constrained setting of a middle-income country. Although MEOWS is a well-known tool to recognize clinical deterioration in pregnant women,^41^ its application in Brazilian public maternity hospitals was novel for many hospitals.^31^ During the COVID-19 pandemic, the Brazilian Ministry of Health recommended using the tool in all public hospitals assisting pregnant women.^42^

Our intervention introduced novel combinations and applications of existing QI tools and approaches. While some hospitals had previously utilized the MEOWS, our initiative innovated by linking MEOWS with care bundles and mandating MEOWS calculation during all transitions of care and following bundle implementation. This comprehensive integration of MEOWS represented a unique approach to enhancing patient safety and standardizing care practices. This BTS design linked a process that reliably identified patients at risk of life-threatening conditions to a reliable mechanism to deliver necessary care quickly. The “4Rs” system enabled all participants, not just the physicians, to respond when the clinical team identified a woman at risk; this meant that nursing teams in the participating hospitals could trigger and implement the bundles—an unusual role in the Brazilian setting.

Although many hospitals have postpartum hemorrhage protocols, evidence suggests that the mere presence of these protocols does not reduce bleeding-related deaths.^43^ In our initiative, the use of QI and training to improve the reliability of the application of actions for preventing, detecting, and implementing bundles to treat hemorrhage-related deaths was associated with a 72.9% reduction in iMMR. A similar impact on maternal hemorrhage was reported recently by Gallos et al.,^44^ who saw a significant decrease in postpartum hemorrhage in an intervention group of African hospitals that received a clinical bundle implemented with a package of activities that included training, local champions, and audit and feedback; mortality was not reported. Other studies showed a direct correlation between adherence to bundles and improved maternal outcomes and provider performance.^36^

The early identification of sepsis, even before a definitive diagnosis, is crucial in preventing maternal mortality,^45^ mainly as maternal sepsis definitions are controversial.^46^^–^^48^ While sepsis is reported as the third leading cause of death worldwide,^2^ infection is responsible for nearly half (46%) of all facility-based maternal deaths in Brazil.^49^ This BTS showed that early detection through the MEOWS, together with the introduction of the suspicion of sepsis bundle, was associated with a 100% decrease in sepsis-associated maternal deaths, reinforcing the recommendation of early detection and treatment^50^ (i.e., application of the sepsis bundle within an hour of suspicion of sepsis), rather than waiting for sepsis to be confirmed.

After the QI intervention, iMMR due to HDPs did not change from the baseline values despite adaptive changes to the implementation design (e.g., additional clinical sessions, reinforcements in quality training, adjustments, and customization of the HDP bundle for each maternity unit setting). We also did not observe improvement in bundle compliance for HDP. Failure to reduce maternal mortality due to HDP include poor implementation of the HDP bundle, the inadequacy of the HDP-specific bundle, the clinical condition of patients in this group (age, comorbidities, cardiovascular risk, and others), structural issues (lack of required equipment and medications and different concentrations of magnesium sulfate among hospitals making standardization more difficult), and poor antenatal care (patients arriving at the hospitals with advanced HDP). While the California Pregnancy-Associated Mortality Review Committee in the United Sates attributed 65% of HDP-related deaths to missed diagnoses and ineffective care,^51^ our findings suggest that existing clinical care bundles and QI interventions and training to deploy those bundles were insufficient to rescue women presenting with HDP. Other QI interventions using the toolkit approach have successfully reduced eclampsia and morbidity due to HDP,^52^ but the impact of this approach on mortality was not reported. Improving prenatal care, standardization of medication doses among hospitals, and incorporating new implementation strategies or complementary clinical activities may be necessary to decrease HDP-related deaths in Brazil.

Furthermore, the absence of significant changes in maternal mortality due to HDP despite reductions in deaths from postpartum hemorrhage and sepsis warrants further analysis. While our intervention focused on improving recognition and management through care bundles, excluding the onset of labor and mode of delivery as a component of the HDP care bundle may have limited its effectiveness. However, decisions regarding the mode and timing of delivery are complex, often influenced by a combination of clinical, provider, and patient preferences, as well as institutional practices. In our setting, the intervention did not address these factors directly, which may have contributed to the lack of observed improvement in HDP-related mortality. This highlights the need for future iterations of care bundles to incorporate interventions, such as early delivery protocols, that directly address the clinical progression of HDP.

Finally, we highlight that we undertook this QI initiative during the COVID-19 pandemic. Despite the adverse context, the initiative achieved sufficient engagement of the obstetric teams to continue actively participating in the adapted QI initiative. Teams continued to attend virtual learning sessions and undertake activities in their maternity units. The improved outcomes for hemorrhage and sepsis were sustained despite an increase in overall iMMR attributed to COVID-19 infection.^12^^,^^13^ The second wave of COVID-19 coincided with the final months of this BTS; after March 2021 COVID-19 became the leading cause of maternal death, obscuring the impact of the QI work performed by this BTS. The adjustments to deliver the BTS virtually during the pandemic provided a potential model for remote QI implementation and training.

### Limitations

Although our work showed a significant decrease in iMMR for postpartum hemorrhage and sepsis, the results should be interpreted cautiously. The hospitals selected for the study had considerable variations in baseline performance and significant variations in the number and values of iMMR. Variations in baseline iMMR and low actual numbers of maternal deaths per month in each hospital did not allow for rigorous subgroup analysis. The hospitals that actively participated in bundle deployment likely contributed disproportionately to reported improvements in mortality for postpartum hemorrhage and sepsis in the aggregated results. In addition, some hospitals already had high-quality maternity programs with low or no maternal deaths, providing a minimal opportunity for contribution to overall improved iMMR. However, these hospitals provided significant opportunities for a study of positive deviance, supporting ideas for improvement across the learning collaborative.

A more rigorous selection of intervention hospitals and inclusion of a matched group of hospitals that did not receive the intervention would have increased our confidence that the reductions in mortality from postpartum hemorrhage and sepsis were directly attributable to the QI and training strategies that we deployed. However, we know of no other environmental or policy interventions that could be attributed to our observed results.

While we focused on iMMR as an aggregate outcome, the lack of granular data on presenting cause-specific cases limits our ability to generate additional insights. This underscores the importance of future QI initiatives adopting more comprehensive data collection frameworks, including systematic collection and analysis of case tracking, clinical intervention timing, and associated outcomes. These data would enable more robust evaluations of intervention effectiveness and help identify critical areas for further refinement. Additionally, while the impact of race and ethnicity are essential factors in determining maternal outcomes in Brazil,^12^ as it is worldwide, we were not able to discern differences in maternal outcomes based on race in this set of hospitals.

The absence of data on perinatal outcomes, such as fetal loss and newborn mortality, also represents a limitation of this study, avoiding having a broader impact of these conditions on the maternal-newborn dyad and restricting the scope of our findings. Future QI initiatives should prioritize the systematic collection of perinatal data to provide a more comprehensive evaluation of QI interventions.

## CONCLUSION

A combination of implementation approaches consisting of QI, Job Instruction, equity and antiracism training, care bundles, and MEOWS was delivered using a Breakthrough Series Collaborative in a group of public hospitals in Brazil to improve the reliability of evidence-based practices that are known to reduce pregnancy-related maternal deaths. We used a QI approach along the peripartum patient journey to deliver a sequential set of clinical surveillance and response activities (the “4Rs”) and ensure reliable implementation of evidence-based care bundles. These activities were linked to a significant decrease in aggregate mortality for 2 of 3 targeted life-threatening conditions in participating public hospitals.

Furthermore, during the implementation and post-implementation periods, deaths related to life-threatening conditions either remained stable or decreased even further despite the excess mortality associated with the COVID-19 pandemic. The scalability of our approach must be further tested using robust evaluation designs in greatly expanded settings and contexts. If replicated in these contexts, this QI implementation and clinical management approach may inform expanded national and global policies and strategies to reduce maternal mortality in LMICs.

## Notes

**Peer Reviewed**
